# Experimental dataset of nanoporous GaN photoelectrode supported on patterned sapphire substrates for photoelectrochemical water splitting

**DOI:** 10.1016/j.dib.2019.104433

**Published:** 2019-08-28

**Authors:** Dongjing Li, Jianghua Liu, Yang Wang, Aixia Wu, Ruolin Ruan, Zeping Li, Zhimou Xu

**Affiliations:** a School of Electronic Information and Engineering, Hubei University of Science and Technology, Xianning 437005, China; b School of Optical and Electronic Information, Huazhong University of Science and Technology, Wuhan 430074, China

**Keywords:** Gallium nitride (GaN), Photoelectrode, Patterned sapphire substrate (PSS), Anodic aluminum oxide (AAO), Photoelectrochemical water splitting

## Abstract

GaN is one of the most promising materials for high PEC efficiency to produce clean, renewable hydrogen in an ecofriendly manner (Ebaid et al., 2015; Kamimura et al., 2017; Yang et al., 2018; Ohkawa et al., 2013). Trough assays of nanoporous gallium nitride (GaN) photoelectrode, we recently demonstrated an improved PEC efficiency and photocurrent density of nanoporous GaN photoelectrode by 470% times with respect to planar counterpart (Li et al., 2019). Here, we report original data acquired under UV–visible spectrometer, X-ray diffraction (XRD), room temperature PL measurements and PEC measurements, based on the characterization of different sapphire substrate, different GaN samples and different GaN photoelectrodes. The optical properties and photoelectrochemical properties of the corresponding samples and possible mechanisms are presented, which is freely available (Li et al., 2019). The data can be valuable for researchers interested in photoelectrochemical water splitting, as well as to researchers developing fabrication of nanoporous photoelectrode. For more insight please see the research article “A nanoporous GaN photoelectrode on patterned sapphire substrates for high-efficiency photoelectrochemical water splitting”, https://doi.org/10.1016/j.jallcom.2019.06.234.

**Table:** Specifications Table

Subject	Chemical engineering
Specific subject area	Photoelectrochemical water splitting
Type of data	Table and figure
How data were acquired	scanning electron microscope (SEM, FEI, Nova 450), UV–visible spectrometer (Varian Cary 500), X-ray diffraction (XRD) (D8, Brucker), room temperature photoluminescence (PL) measurements using a 325 nm He–Ne laser with a 0.75 m monochromator, a potentiostat (Gamry Reference 3000).
Data format	Raw and analyzed
Parameters for data collection	The morphologies of the GaN samples were measured adopting the scanning electron microscope (SEM, FEI, Nova 450). The optical absorbance and reflectance of different GaN samples were characterized using UV Vis (Varian Cary 500). The characterization of PL spectra and XRD give an insight into the improved crystalline quality of samples using XRD (D8, Brucker) and room temperature PL measurements with a 325 nm He–Ne laser with a 0.75 m monochromator. The PEC measurements were carried out in the 1 M NaOH electrolyte with pH = 13.6 in a UV-transparent quartz cell with a customized three-electrode configuration, wherein the GaN photoelectrode, Pt wire and Ag/AgCl (saturated KCl) were used as a working electrode, a counter electrode and a reference electrode, respectively.
Description of data collection	The designed experiments included: fabrication of patterned sapphire substrate (PSS) and gallium nitride (GaN) epitaxial growth, preparation of anodic aluminum oxide (AAO) membrane, transfer of AAO membrane, fabrication of nanoporous GaN. PEC cell measurements.
Data source location	Hubei University of Science and Technology, Xianning, China
Data accessibility	The raw data files are hosted on the public repository of Mendeley Data [6] Li, Zeping (2019), “Experimental dataset on nanoporous GaN photoelectrode on patterned sapphire substrates for high-efficiency photoelectrochemical water splitting”, Mendeley Data, v2 https://doi.org/10.17632/99cb2txmbm.2. The other data is with this article.
Related research article	Z. P. Li et al. “A nanoporous GaN photoelectrode on patterned sapphire substrates for high-efficiency photoelectrochemical water splitting”. Journal of Alloys and Compounds, 2019. https://doi.org/10.1016/j.jallcom.2019.06.234.

**Table:** 

**Value of the data** • The data can serve as a reference to develop a single-step top-down method using the AAO mask for fabrication of the nanoporous GaN photoelectrode grown on PSS. • The shared data can be useful to fabricate the nanoporous structure conveniently and economically. • This work will pave the way towards low-cost and mass production of nanoporous GaN photoelectrode for efficient solar water splitting.

## 1 Data

**Fig. 1:**
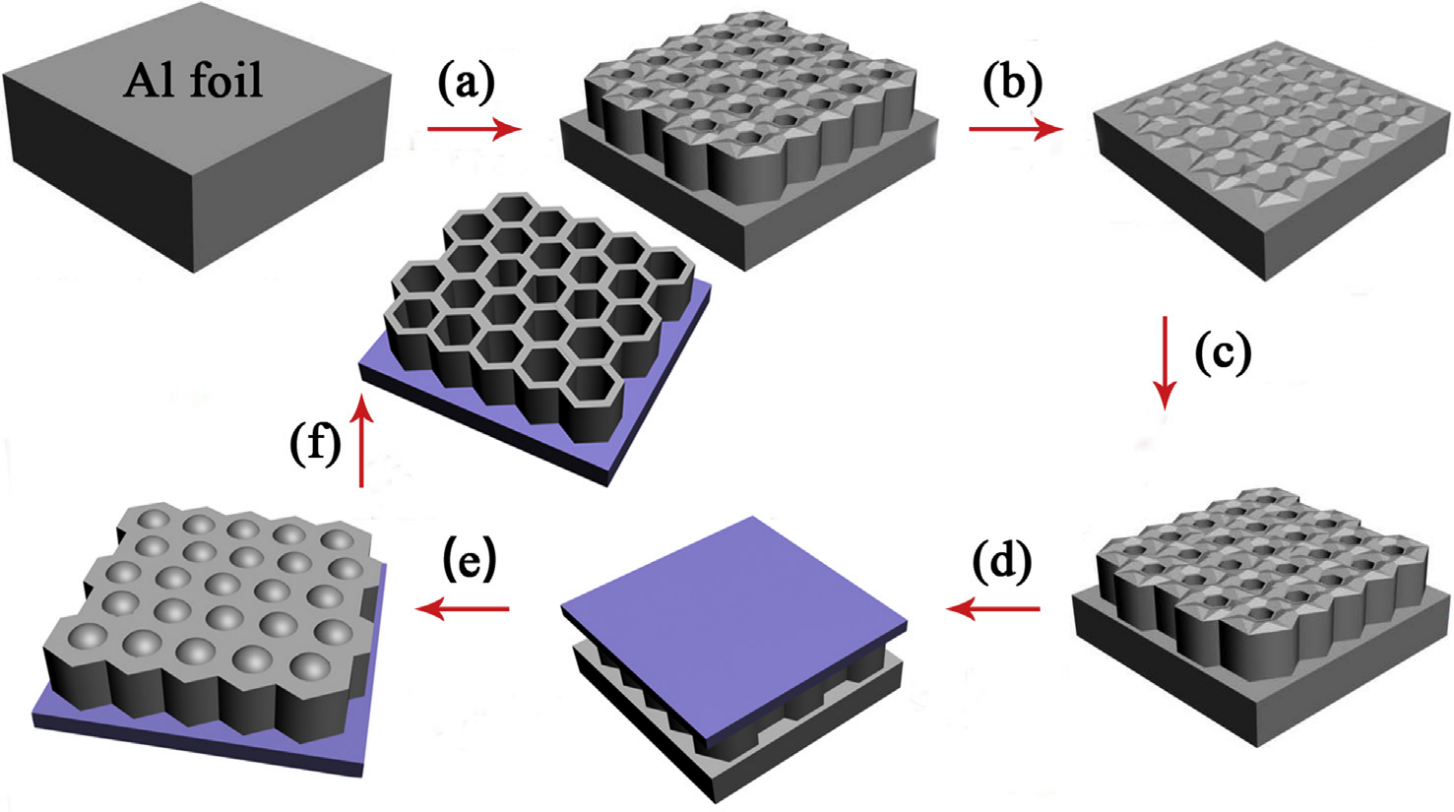
The schematic diagram of preparation process for AAO membrane, (a) first anodization, (b) dissolving of the irregular oxide layer, (c) second anodization, (d) PMMA film was spin-coated, (e) removing of Al substrate, (f) pore-widening and pore-opening.

**Fig. 2:**
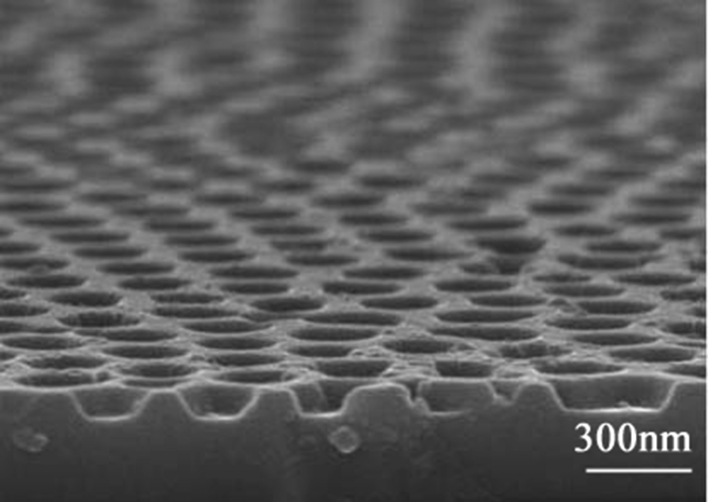
A SEM image of the nannoporous GaN with shallowly etched holes.

**Fig. 3:**
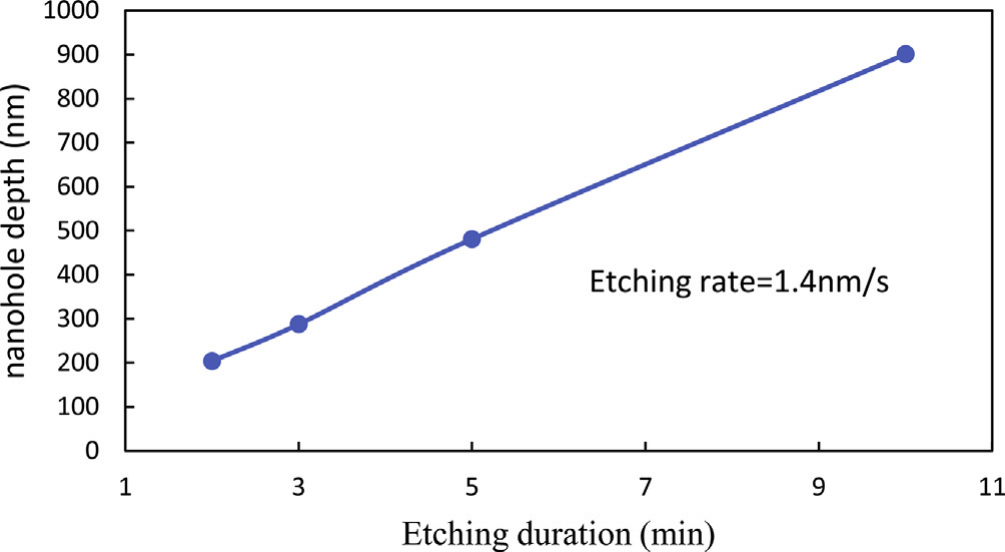
The evolution curve of nannoporous GaN depth as a function of etching duration.

**Fig. 4:**
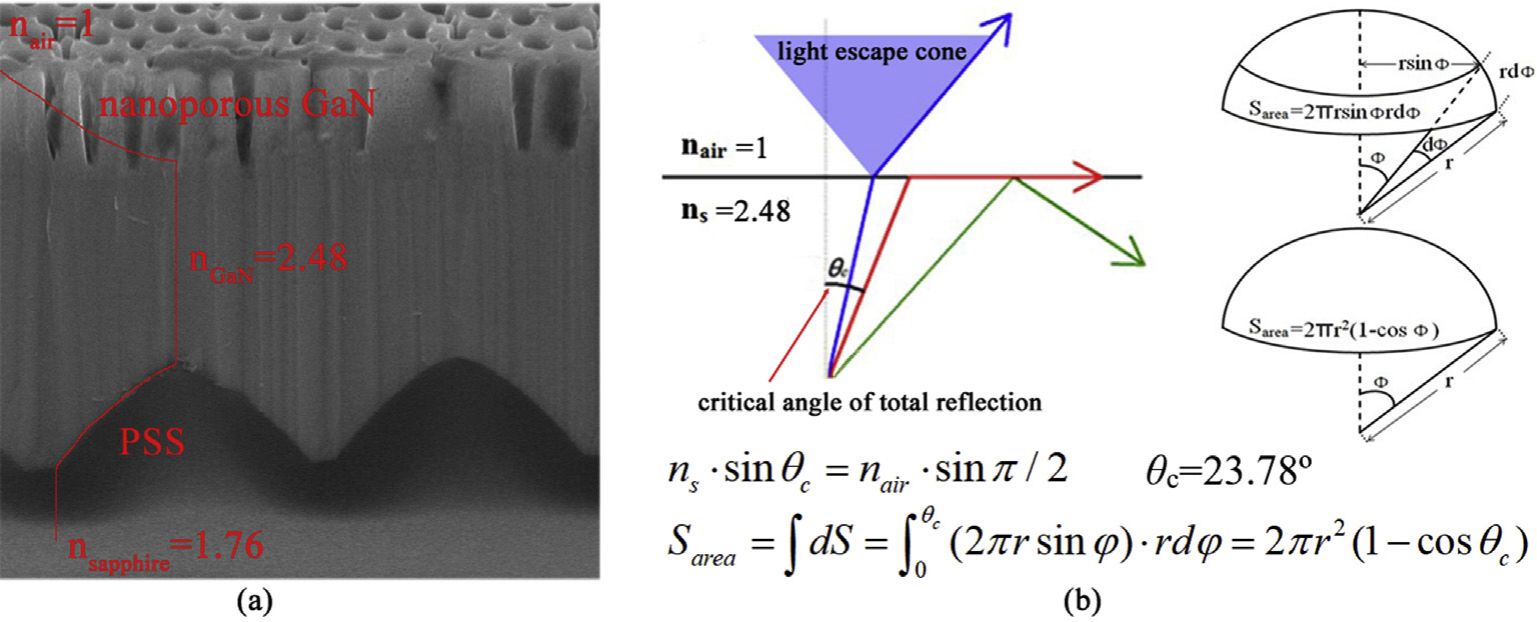
(a) The schematic diagram of the effective refractive index and (b) the light escape cone in the nanoporous GaN.

**Fig. 5:**
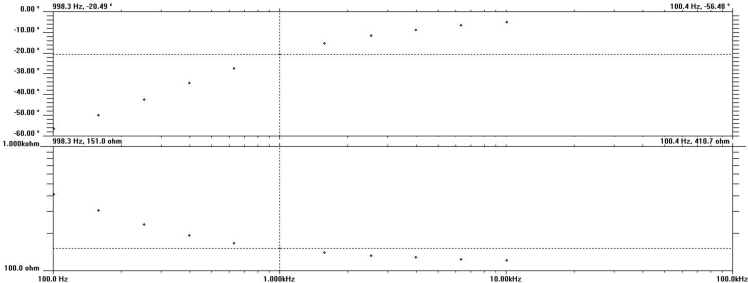
A curve of impedance as a function of frequency.

The shared data are recordings from preparation and characterization process of nanoporous GaN photoelectrode supported on patterned sapphire substrates for high-efficiency photoelectrochemical water splitting [1], [2], [3], [4], [5]. Fig. 1 shows the schematic diagram of preparation process for AAO mask. The morphologies of the GaN samples were measured adopting SEM (Fig. 2). Fig. 3 shows the evolution curve of nannoporous GaN depth as a function of etching duration using ICP dry etching. The nanoporous structure in GaN caused the decrease of the effective refractive index as shown in Fig. 4(a), and the volume of the light escape cone was calculated as shown in Fig. 4(b). The characterization of PL spectra and XRD give an insight into the improved crystalline quality of samples. Table 1 shows the resulting raw data for PL spectra of different GaN samples, and Table 2 shows the resulting raw data for XRD rocking curves for (002) and (102) plane of different GaN samples. The optical absorbance and reflectance of different GaN samples were characterized using UV Vis. Table 3 shows the resulting raw data for optical absorbance and reflectance spectra of different GaN samples. PEC cell measurements were used to characterize the photoelectrochemical properties of different GaN photoelectrodes, wherein the EIS measurement was carried out at the applied frequencies of range from 10 kHz to 0.1 Hz as shown in Fig. 5. The resulting raw data are shown in Tables 4,5,6,7. Table 4 shows raw data for current density-potential plots of different GaN photoelectrodes for dark and light-on photocurrent. Table 5 shows raw data for transient current density-time plots of different GaN photoelectrodes for dark and light-on photocurrent. Table 6 shows raw data for Nyquist plots of different GaN photoelectrodes. Table 7 shows raw data for current density-time curves for stability assessment of different GaN photoelectrodes and IPCE spectra of different GaN photoelectrodes.

The above-mentioned raw data set in tables is hosted on the public repository of Mendeley Data [6].

## 2 Experimental design, materials, and methods

The preparation of anodic aluminum oxide (AAO) membrane was performed in a self-made instrument. As shown in Fig. 1, before anodizing of a 0.2 mm-thick Al foil (99.999% in purity), annealing, cleaning and electrochemical polishing were carried out. The first anodization was performed in oxalic acid at 1 °C and 120 V for 4 h, followed by dissolving of the irregular oxide layer using mixture of chromic acid and phosphoric acid for 8 h. The second anodization was performed in the same conditions for 4 minutes. Finally, a polymethyl methacrylate (PMMA) film was spin-coated on AAO surface of the as-prepared sample, followed by removing of Al substrate in a saturated copper chloride, pore-opening and pore-widening in phosphoric acid solution in turn [7], [8].

To fabricate the nanoporous structure in GaN, a single-step top-down etching approach is developed using an AAO mask. During the ICP etching, the plasmas of ICP system passed through the hole channel of AAO and etched the exposed GaN surface on the bottom of AAO holes. The morphologies of the GaN samples were measured adopting the scanning electron microscope (SEM, FEI, Nova 450) at acceleration voltage of 10 kV and magnification of 100,000 times. Under the shallowly etching, the nannoporous GaN shows steep holes, as shown in Fig. 2.

The method for growing the photoelectrode on the substrates was presented in the research article “A nanoporous GaN photoelectrode on patterned sapphire substrates for high-efficiency photoelectrochemical water splitting” Li et al., 2019. Wherein, ICP (Oxford Plasmalab 100) dry etching is carried out at 3.5 mTorr pressure, 150 W RF power and 600 W coil power, with Cl_2_/BCl_3_ (15 sccm/60 sccm) gases for fabrication of nanoporous GaN. The etching duration determines the depth of nannoporous GaN, as shown in Fig. 3.

For the nanoporous GaN, the nanoporous structure caused the decrease of the effective refractive index (Fig. 4(a)), the volume of the light escape cone was calculated as shown in Fig. 4(b).

To assess photoelectrochemical properties of different GaN photoelectrodes, PEC cell measurements were carried out. Before PEC cell measurements, Ti/Au (10/150 nm) films were evaporated on the top of GaN edge as contact electrodes via e-beam evaporation. Subsequently, the photoelectrode was sealed with PVC tape and epoxy to define the PEC working area and insulation area from the electrolyte. The PEC measurements were carried out in the NaOH electrolyte with pH = 13.6 in a UV-transparent quartz cell with a three-electrode configuration, wherein the GaN photoelectrode, Pt wire and Ag/AgCl (saturated KCl) were used as a working electrode, a counter electrode and a reference electrode, respectively. During water splitting, a 300 W Xe lamp (Newport) was used to simulate one sun illumination (under AM 1.5 illumination at 100 mW cm^−2^), and an air mass 1.5G filter (Newport) was used to filter 97% ultraviolet (UV) light for reduction of photocorrosion to GaN photoelectrode. As shown in Fig. 5, the EIS measurement was carried out at the applied frequencies of range from 10 kHz to 0.1 Hz under illumination at an applied potential of 1.23 V vs. RHE.
